# Phosphorylated Akt1 expression is associated with poor prognosis in cutaneous, oral and sinonasal melanomas

**DOI:** 10.18632/oncotarget.26458

**Published:** 2018-12-18

**Authors:** Ciro Soares, Thayná Melo de Lima Morais, Roman Carlos, Fernanda Viviane Mariano, Albina Altemani, Maria Goretti Freire de Carvalho, Marcelo Brum Corrêa, Rodrigo Ribas Dias dos Reis, Luciana Schultz Amorim, Oslei Paes de Almeida, Jacks Jorge

**Affiliations:** ^1^ Department of Oral Diagnosis, Area of Pathology, Piracicaba Dental School, University of Campinas, Piracicaba, São Paulo, Brazil; ^2^ Pathology Division, Centro Clínico de Cabeza y Cuello/Hospital Herrera Llerandi, Guatemala City, Guatemala; ^3^ Department of Pathology, Faculty of Medical Sciences, University of Campinas, Campinas, São Paulo, Brazil; ^4^ Private Pathology Service, Natal, Rio Grande do Norte, Brazil; ^5^ Head and Neck Surgery Department - Oncology Center (CEON), Fornecedores de Cana Hospital, Piracicaba, São Paulo, Brazil; ^6^ Oncology Surgery Department - Cancer Center (CECAN), Santa Casa Hospital, Piracicaba, São Paulo, Brazil; ^7^ Institute of Pathological Anatomy, Piracicaba, São Paulo, Brazil

**Keywords:** cutaneous melanomas, mucosal melanomas, p-Akt1, immunohistochemistry, prognosis

## Abstract

Melanomas are highly aggressive tumours derived from melanocytes, which occur most commonly in the skin. Occasionally, these tumours may appear in oral and sinonasal mucous membranes. In this study, we performed a comparative analysis of the Phosphorylated Akt1 (p-Akt1) expression in 144 patients affected by cutaneous (CM), 34 oral cavity (OM), and 31 sinonasal melanomas (SNM). Similar to the metastatic cutaneous melanomas, p-Akt1 was overexpressed in 17/34 of the oral cavity and 20/31 of the sinonasal melanomas. In addition, the p-Akt1-nuclear expression was associated with poorer cancer-specific survival in cutaneous (*P* < .0001), oral (*P* < .0001), and sinonasal (*P* = .001) melanomas. Multivariate analysis showed p-Akt1 to be an independent prognostic marker in oral (*P* = .041) and sinonasal (*P* < .0001) melanomas patients. In conclusion, p-Akt1 overexpression is an independent prognostic marker in mucosal melanomas and is significantly up-regulated in sinonasal melanomas. As both mucosal and metastatic cutaneous melanomas showed high frequency of p-Akt1 expression, these findings suggest that mucosal melanomas have a biological behaviour, similar to the aggressive cutaneous melanomas.

## INTRODUCTION

Cutaneous melanomas represent about 1.6% of all cancers, and discreet advances in their treatment have been made over the last decades. Nevertheless, this tumour still remains deathly, especially the metastatic disease [1]. It is estimated that melanomas with lymph nodal metastases are responsible for 59,782 global deaths, with mean overall survival rate of 13% in 5 years [2]. Controlling the advanced-stage disease is the major problem for the treatment of melanomas. Thus, for the development of targeted therapies, making progress in understanding the molecular factors that influence the aggressiveness of the metastatic melanomas is essential.

Until now, comparative studies with melanomas from different anatomic sites that assessed the potential differences and similarities between these tumours are scarce [3–6]. Overall, the etiologic factors and the clinical and biological behaviour of cutaneous melanomas are very distinct from the other melanomas [7, 8]. For example, mucosal melanomas constitute a particular subset of all melanomas characterized by high-aggressive behaviour, tendency to metastasize and consequently association with marked worse prognosis. Sinonasal melanomas in some series have presented a high-mortality index (near 100%) [8, 9]. Clinicopathological parameters determine the prognosis and staging of the melanomas from different anatomic sites [10].

The use of biological markers, especially by immunohistochemistry, for predict prognosis is still poorly explored in mucosal melanomas. The serine/threonine protein B Kinase, known as Akt, has an oncogenic function in several tissues by regulating cell proliferation, migration and invasion [11]. The overexpression of p-Akt1 is correlated with adverse outcome in breast [12], gastric [13] and oesophageal squamous cell carcinomas [14]. In the context of melanoma, an overgrowing interest in determining as p-Akt1 acts during melanoma progression [15, 16] has been recently observed, and its inhibition has emerged as an interesting targeted therapy for these tumours [17].

Although some studies have demonstrated the role of p-Akt1 in several cancers, including melanoma metastases in a mice model [18], no studies have evaluated the prognostic value of the immunohistochemical expression of p-Akt1 in different subsets of melanomas. In the present study, we evaluated the immunohistochemical expression and the prognostic value of p-Akt1 expression in a large cohort of cutaneous, oral, and sinonasal melanomas.

## RESULTS

### Cutaneous melanomas

The age range of the cohort of 144 patients affected by cutaneous melanomas was from 20 to 88 years old at diagnosis (mean age, 56). The three most common sites were the trunk (64/144), followed by the lower limbs (40/144) and the head and neck area (22/144) (Table 1). The median follow-up period was 62 months, ranging from 14 months to 18 years. The 3- and 5-year cancer-specific survival (CSS) rates were 89.9% and 69.3%, respectively. Detailed clinicopathological data from the cutaneous melanomas is shown in Supplementary Table 1.

**Table 1:** The relationship between clinicopathological characteristics of patients with cutaneous melanomas, p-Akt1 expression and cancer-specific survival

Factors	Sample *n* (%)	CSS (%)		Univariate *P* (log-rank)	Multivariate	
		3-years	5-years		HR (95% CI)	*P*-value
Age						
<56	78	94.8%	87.6%	**.013 (6.118)**	2.029 (.969–4.250)	.061
≥56	66	82.5%	70.4%			
Ulceration						
Present	61	76.5%	60.3%	**<.0001 (39.745)**	1.569 (.499–4.933)	.441
Absent	83	98.8%	94.4%			
Breslow’s Thickness						
<1.55	72	100%	98.4%	**<.0001 (42.747)**	10.855 (1.101–107.057)	**.041**
≥1.55	72	78.7%	61.7%			
Mitotic rate						
<3	60	98.3%	94.2%	**<.0001 (17.848)**	1.311 (.373–4.606)	.673
≥3	84	82.9%	70.1%			
Clark’s level						
I and II	47	100%	100%	**.002 (9.706)**	.667 (.216–2.063)	.482
III, IV and V	97	84.7%	71.4%			
Distant metastasis						
Present	34	67.5%	41.2%	**<.0001 (62.087)**	2.277 (.512–10.135)	.280
Absent	110	96.3%	92.8%			
AJCC-stage						
*In situ*	15	100%	100%	**<.0001 (71.219)**	1.438 (.650–3.181)	.369
I	51	100%	100%			
II	42	90.4%	80.1%			
III	23	69.3%	55.4%			
IV	13	69.2%	27.7%			
p-Akt1						
High expression	69	80.8%	64.2%	**<.0001 (24.206)**	2.766 (.952–8.038)	.062
Low expression	75	98.6%	94.1%			

CSS – Cancer–specific survival, HR – Hazard Ratio, AJCC – American Joint Committee on Cancer. Bold values indicate statistical significance (*P* < .05).

**Figure 1:**
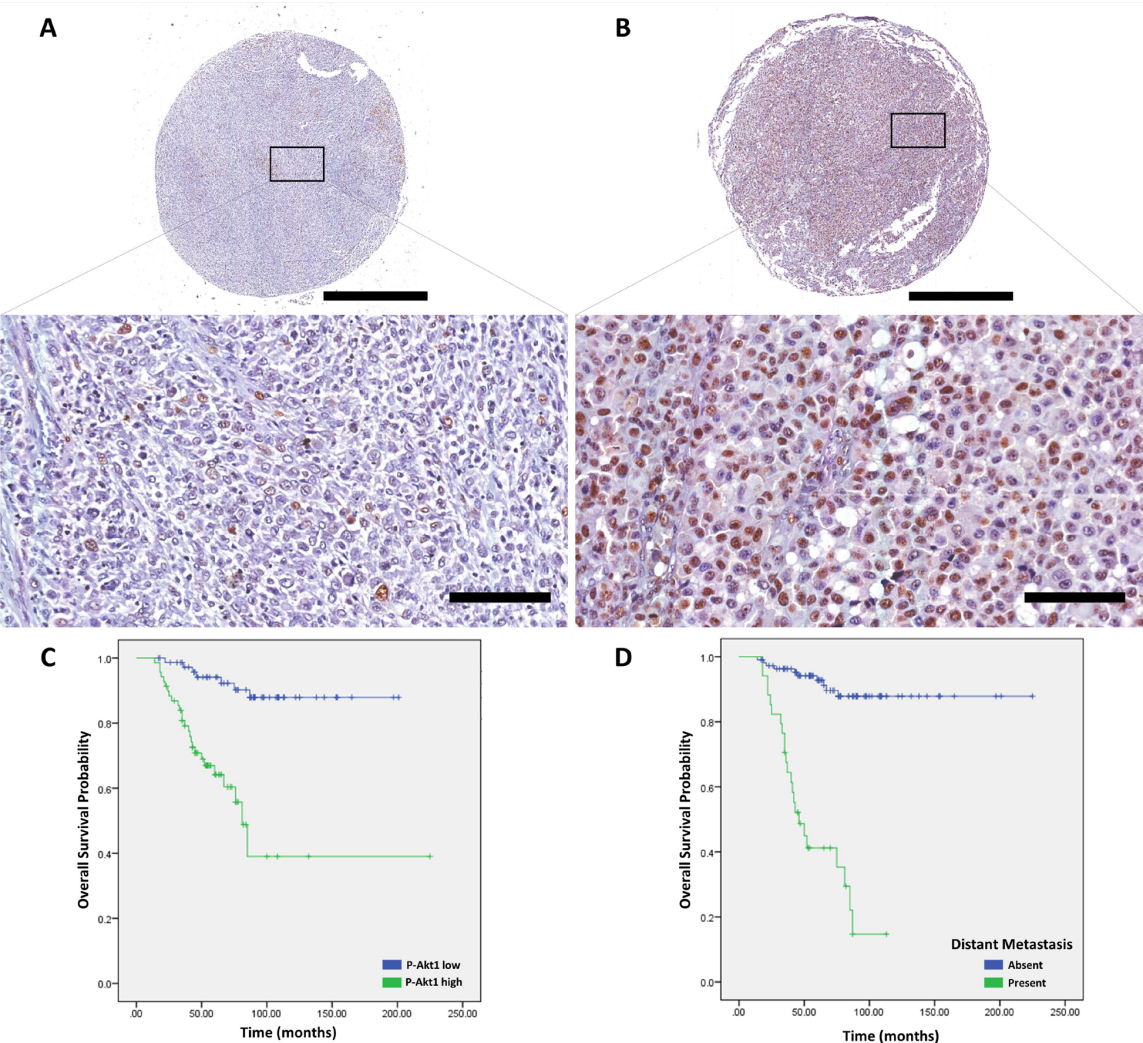
p-Akt1 expression in cutaneous melanomas (**A**) Representative example of non-metastatic cutaneous melanoma in a tissue microarray, which was focally positive for p-Akt1. (**B**) Representative example of metastatic cutaneous melanoma in a tissue microarray, which was strongly positive for p-Akt1. (**C**) The association of p-Akt1nuclear expression and cancer-specific survival in cutaneous melanomas (logrank = 24.206, *P* < .0001). (**D**) The association of metastasis and cancer-specific survival in cutaneous melanomas (log-rank = 62.087, *P* < .0001). The scale bars represent 1 mm (top) and 100 μm (bottom).

Cancer-specific survival (CSS) rates for cutaneous melanomas based on clinicopathological parameters are shown in Table 1. Significant prognostic factors for CSS using a univariate model were Age (*P* = .013), Ulceration (*P* < .0001), Breslow’s Thickness (*P* < .0001), Mitotic rate (*P* < .0001), Clark’s level (*P* = .002), Distant metastasis (*P* < .0001), AJCC-stage (*P* < .0001) and p-Akt1 expression (*P* < .0001) Figure 1; while the unique independent prognostic factor in a multivariate analysis was Breslow Thickness (HR = 10.855, *P* = .041).

### p-Akt1 is an independent prognostic marker in Oral melanomas

For oral melanomas, 34 patients were enrolled in this study, with a mean age of 52 years, and regarding sex, 18 were men and 16 women. The main locations for oral melanomas were the hard palate (15/34), the tooth alveoluls (5/34), and other areas (14/34). The OM clinicopathological data are listed in Supplementary Table 2. The median follow-up period was 23.5 months, ranging from 2 months to 14 years. The 3- and 5-year CSS rates were 49.1% and 35.8%, respectively.

**Figure 2:**
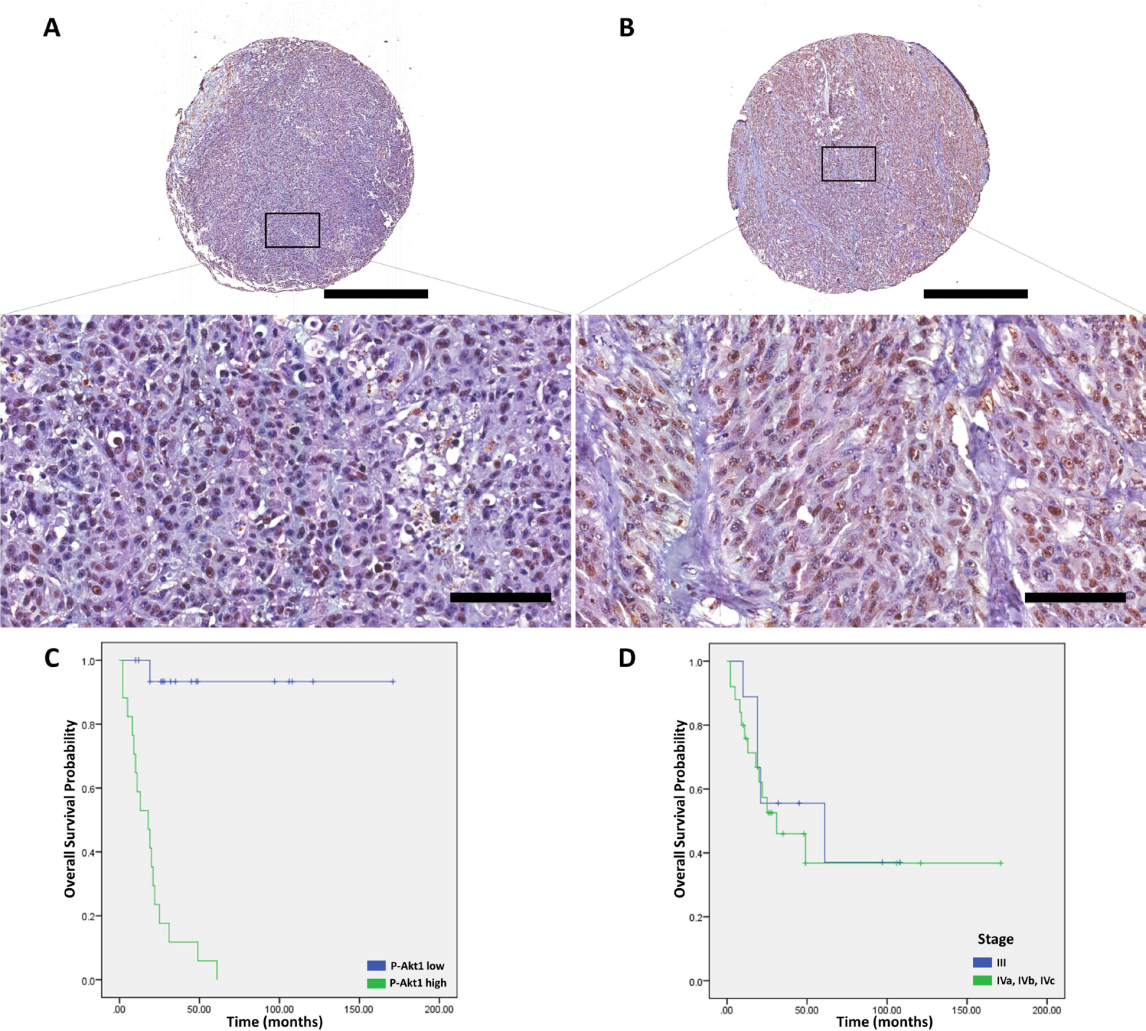
p-Akt1 expression in oral melanomas (**A**) Representative example of oral melanoma in a tissue microarray, which was focally positive for p-Akt1. (**B**) Representative example of oral melanoma in a tissue microarray, which was strongly positive for p-Akt1. (**C**) The association of p-Akt1-nuclear expression and cancer-specific survival in oral melanomas (log-rank = 28.086, *P* < .0001). (**D**) The association of AJCC-stage and cancer-specific survival in oral melanomas (log-rank = .168, *P* = .682). The scale bars represent 1 mm (top) and 100 μm (bottom).

Using the multivariate analysis, the mitotic rate (*P* = .001), presence of vascular invasion (*P* < .0001), neural invasion (*P* = .011), epithelioid cellular morphology (*P* = .001), and p-Akt1 nuclear expression were considered prognostic factors for CSS in oral melanomas (*P* < 0.001, Log-rank = 28.086; Figure 2). More interestingly, in the Cox regression model against established pathological prognostic factors such as mitotic rate, cellular morphology, vascular and neural invasion, p-Akt1 expression demonstrated to be an independent prognostic factor for oral melanomas Table 2 (HR = 11.397, *P* = .041).

**Table 2:** The relationship between clinicopathological characteristics of patients with oral melanomas, p-Akt1 expression and cancer-specific survival

Factors	Sample (%)	CSS (%)		Univariate *P* (log-rank)	Multivariate	
		3-year	5-year		HR (95% CI)	*P*-value
Age						
<47	18 (53.9)	53.8%	38.5%	.926 (.009)	1.407 (.237–8.361)	.707
≥47	16 (46.1)	41.5%	41.5%			
Gender						
Female	16 (46.1)	38.4%	38.4%	.374 (.791)	.718 (.196–2.628)	.617
Male	18 (53.9)	58.2%	38.8%			
Treatment						
Only surgery	22 (64.7)	55.4%	55.4%	.070 (3.273)	.689 (.129–3.674)	.663
Surgery + chemo/radiotherapy	11 (32.4)	38.1%	12.7%			
Anatomical site						
Palate	15 (44.1)	34.2%	34.2%	.113 (4.355)	–	–
Others	19 (55.9)	68.8%	55%			
Mitotic rate						
<1	14 (41.2)	85.1%	85.1%	**.001 (10.116)**	1.372 (.154–12.212)	.777
≥1	20 (58.8)	26.9%	13.5%			
Vascular invasion						
Absent	21 (61.8)	80.4%	58.6%	**<.0001 (18.408)**	1.485 (.191–11.579)	.706
Present	13 (38.2)	0%	0%			
Neural invasion						
Absent	26 (76.5)	42.9%	42.9%	**.011 (6.467)**	4.622 (.640–33.373)	.129
Present	8 (23.5)	15.6%	15.6%			
Cellular morphology						
Epithelioid	22 (64.7)	30.3%	20.2%	**.001 (12.052)**	.927 (.085–10.121)	.951
Non-epithelioid	12 (35.3)	90%	90%			
AJCC-stage						
III	9 (26.5)	55.6%	37%	.682 (.168)	.885 (.211–3.706)	.867
IVa, b and c	25(73.5)	46%	36.8%			
p-Akt1						
High expression	17 (50)	11.8%	5.9%	**<.0001 (28.086)**	**11.397 (1.102–117.855)**	**.041**
Low expression	17 (50)	93.3%	93.3%			

CSS – Cancer–specific survival, HR – Hazard Ratio, AJCC – American Joint Committee on Cancer. Bold values indicate statistical significance (*P* < .05)

### p-Akt1 is an independent prognostic marker in sinonasal melanomas

Regarding the sinonasal melanomas (SNM), out of 31 patients, 15 were women and 16 men of ages between 24 and 82 (mean age, 55). The tumours were located at nasal cavity (41.9%), maxillary sinus (38.7%), and rhinopharynx (19.4%). Detailed clinicopathological data for SNM are shown in Supplementary Table 3. The median follow-up for SNM patients was 30 months, ranging from 3 months to 18.6 years, and the CSS 3-years and 5-years survival rates were 56.9% and 31.8%, respectively.

**Figure 3:**
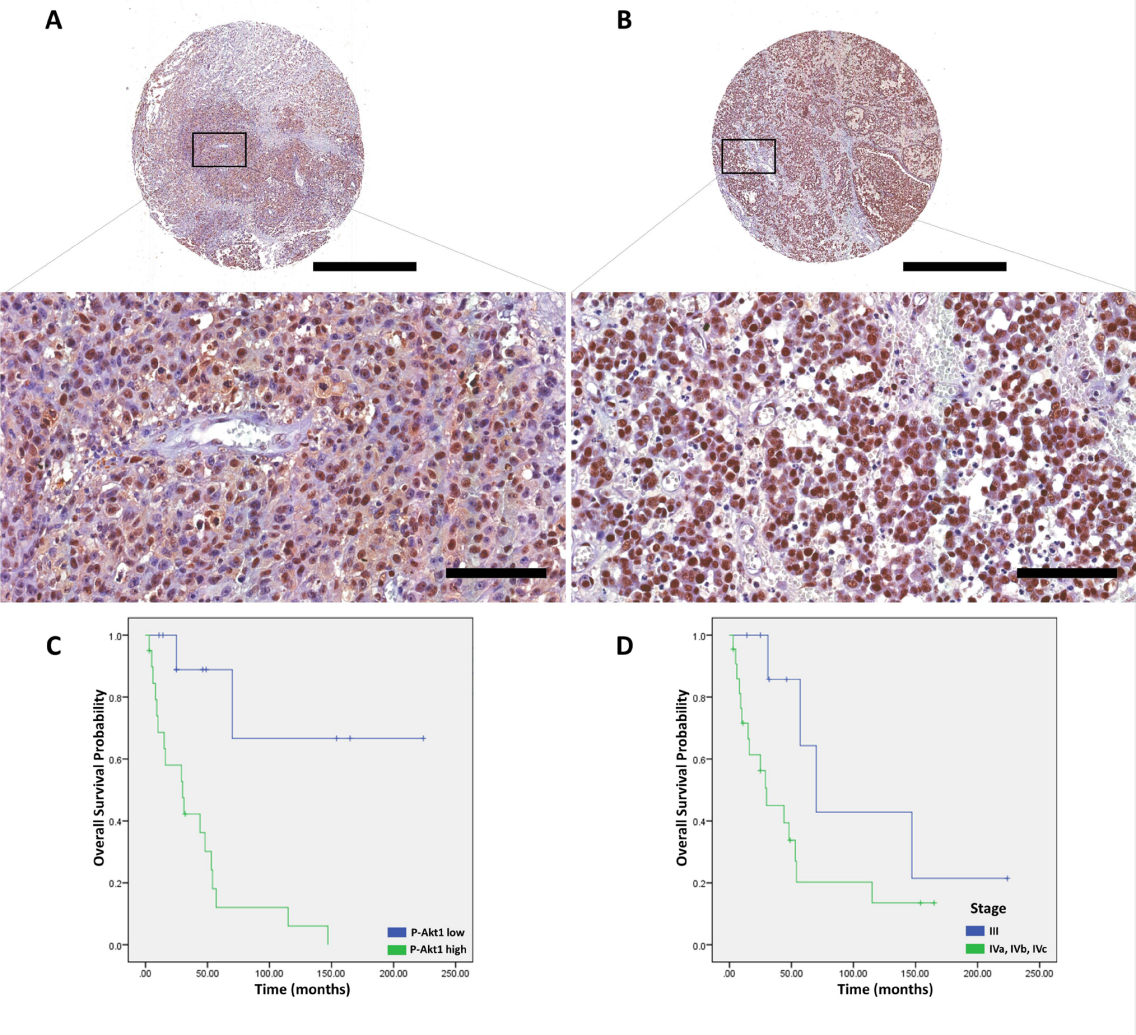
p-Akt1 expression in sinonasal melanomas (**A**) Representative example of sinonasal melanoma in a tissue microarray, which was focally positive for p-Akt1. (**B**) Representative example of sinonasal melanoma in a tissue microarray, which was strongly positive for p-Akt1. (**C**) The association of p-Akt1-nuclear expression and cancer-specific survival in sinonasal melanomas (log-rank = 11.079, *P* = .001). (**D**) The association of AJCC-stage and cancer-specific survival in sinonasal melanomas (logrank = 3.544, *P* = .060). The scale bars represent 1 mm (top) and 100 μm (bottom).

In a univariate model, vascular invasion (*P* = .042) and fusiform cellular morphology (*P* = .001) were associated with poorer CSS for patients with SNM (Table 3). p-Akt1 was associated with poor CSS for SNM in the univariate (*P* = .001, log-rank = 11.079) and multivariate (HR = 65.726, *P* < .0001) models Figure 3. In addition, the clinical grade was also an independent prognostic marker for SNM (HR = 7.351, *P* = .019, Table 3).

**Table 3:** The relationship between clinicopathological characteristics of patients with sinonasal melanomas, p-Akt1 expression and cancer-specific survival

Factors	Sample	CSS (%)		Univariate	Multivariate	
	(%)	3-year	5-year	*P* (log-rank)	HR (95% CI)	*P*-value
Age						
<58	16 (51.6)	60.6%	30.3%	.701 (.147)	1.935 (.479–7.820)	.354
≥58	15 (48.4)	51.3%	38.5%			
Gender						
Female	15 (48.4)	67%	41.9%	.319 (.994)	–	–
Male	16 (51.6)	49.2%	25.3%			
Treatment						
Only surgery	13 (41.9)	63.5%	25.4%	.604 (.268)	.473 (.132–1.694)	.250
Surgery + chemo/radiotherapy	17 (54.8)	52.7%	36.1%			
Anatomical site						
Nasal cavity	13 (41.9)	56.6%	45.3%	.239 (2.860)	–	–
Others	18 (58.1)	50%	0%			
Mitotic rate						
<1	20 (64.5)	60.2%	50.2%	.401 (.707)	.361 (.103–1.260)	.110
≥1	11 (35.5)	54.5%	10.9%			
Vascular invasion						
Absent	22 (70.9)	63.9%	45.7%	**.042 (4.151)**	.280 (.072–1.080)	.065
Present	9 (28.1)	44.4%	11.1%			
Neural invasion						
Absent	27 (87.1)	63.1%	38.5%	.072 (3.230)	3.223 (.616–16.857)	.166
Present	4 (12.9)	25%	0%			
Cellular morphology						
Epithelioid	9 (29.1)	100%	62.5%	**.001 (17.600)**	.582 (.325–1.040)	.068
Fusiform	13 (41.9)	15.4%	7.7%			
AJCC-stage						
III	9 (28.1)	85.7%	64.3%	.060 (3.544)	**7.351 (1.392–38.821)**	**.019**
IVa, b and c	22 (70.9)	45%	20.2%			
p-Akt1						
High expression	20 (64.5)	42.2%	12.1%	**.001 (11.079)**	**65.726 (6.491–665.549)**	**<.0001**
Low expression	11 (35.5)	88.9%	88.9%			

CSS – Cancer–specific survival, HR – Hazard Ratio, AJCC – American Joint Committee on Cancer. Bold values indicate statistical significance (*P* < .05)

### p-Akt1 expression is associated with clinicopathological parameters in melanomas

In the cutaneous melanomas patients, p-Akt1 showed positive association with ulceration (*P* < .0001), growth phase (*P* < .0001), Breslow’s Thickness (*P* < .0001), mitotic rate (*P* = .003), Clark’s Level (*P* < .0001), metastasis (*P* = .008), AJCC-stage (*P* < .0001), and recurrence (*P* < .0001), as shown in Table 4. For oral melanomas, p-Akt1 expression showed association with vascular invasion (*P* = .001), mitotic rate (*P* < .0001), and epithelioid cellular morphology (*P* < .0001), as displayed in Table 5. In addition, in the SNM cohort (Table 6), vascular invasion (*P* = .008), mitotic rate (*P* = .023), and fusiform cellular morphology (*P* = .009) were significantly associated with the positive p-Akt1-nuclear expression.

**Table 4:** The relationship between p-Akt1 nuclear expression and clinicopathological characteristics of 144 patients with cutaneous melanomas

Clinicopathological characteristics	p-Akt1 low	p-Akt1 high	*P*-value
**Age (<56/≥56)**	44/31	34/35	.259
**Gender (Female/Male)**	39/36	32/37	.500
**Site (Trunk/Head and neck/Upper limbs/Lower Limbs)**	33/11/9/22	31/11/9/18	.975
**Ulceration (Absent/Present)**	63/12	20/49	**<.0001**
**Growth phase (Radial/Vertical)**	40/35	15/54	**<.0001**
**Breslow’s Thickness (<1.55/≥1.55)**	59/16	13/56	**<.0001**
**Mitotic rate (<3/≥3)**	40/35	20/49	**.003**
**Clark’s Level (I and II/III, IV and V)**	40/35	7/62	**<.0001**
**Metastasis (Absent/Present)**	64/11	46/23	**.008**
**AJCC-stage (*in situ*/I/II/III/IV)**	15/39/10/8/3	0/12/32/15/10	**<.0001**
**Recurrence (Absent/Present)**	61/14	26/43	**<.0001**

**Table 5:** The relationship between p-Akt1 nuclear expression and clinicopathological characteristics of 34 patients with oral melanomas

Clinicopathological characteristics	p-Akt1 low	p-Akt1 high	*P*-value
**Age (<47/≥47)**	8/9	10/7	.492
**Gender (Female/Male)**	8/9	8/9	1.000
**Site (Palate/alveolus/others)**	6/2/9	9/3/5	.379
**Treatment (only surgery/surgery plus chemo-radiotherapy)**	13/4	9/7	.151
**Vascular invasion (Absent/Present)**	15/2	6/11	**.001**
**Neural invasion (Absent/Present)**	14/3	12/5	.419
**Mitotic rate (<1/≥1)**	12/5	2/15	**<.0001**
**Necrosis (Absent/Present)**	14/3	10/7	.132
**Cellular morphology (Non-epithelioid/Epithelioid)**	12/5	0/17	**<.0001**
**Recurrence (No/Yes)**	14/3	13/4	.671

**Table 6:** The relationship between p-Akt1 nuclear expression and clinicopathological characteristics of 31 patients with sinonasal melanomas

Clinicopathological characteristics	p-Akt1 low	p-Akt1 high	*P*-value
**Age (<58/≥58)**	5/6	11/9	.611
**Gender (Female/Male)**	7/4	8/12	.208
**Site (Nasal Cavity/Maxillary sinus/Rhinopharynx)**	6/5/0	7/7/6	.126
**Treatment (only surgery/surgery plus chemo-radiotherapy)**	5/6	8/11	.741
**Vascular invasion (Absent/Present)**	11/0	11/9	**.008**
**Neural invasion (Absent/Present)**	11/0	16/4	.112
**Mitotic rate (<1/≥1)**	10/1	10/10	**.023**
**Necrosis (Absent/Present)**	8/3	10/10	.220
**Cellular morphology (Epithelioid/Fusiform/Plasmacytoid-Undifferentiated)**	6/2/3	3/11/6	**.009**
**Recurrence (No/Yes)**	7/4	12/8	.842

### p-Akt1 expression is higher in metastatic cutaneous melanomas and mucosal melanomas than in non-metastatic cutaneous melanomas

p-Akt1 expression was tested in a large cohort of cutaneous, oral, and sinonasal melanomas, and it was expressed in tumour cells and occasionally in inflammatory cells surrounding the tumour. We also compared the scores of p-Akt1-positive nuclei into four groups: (a) primary (cutaneous melanoma) without metastatic history; and (b) primary (cutaneous melanoma) with metastatic history, (c) oral melanomas, and (d) sinonasal melanomas.

**Figure 4:**
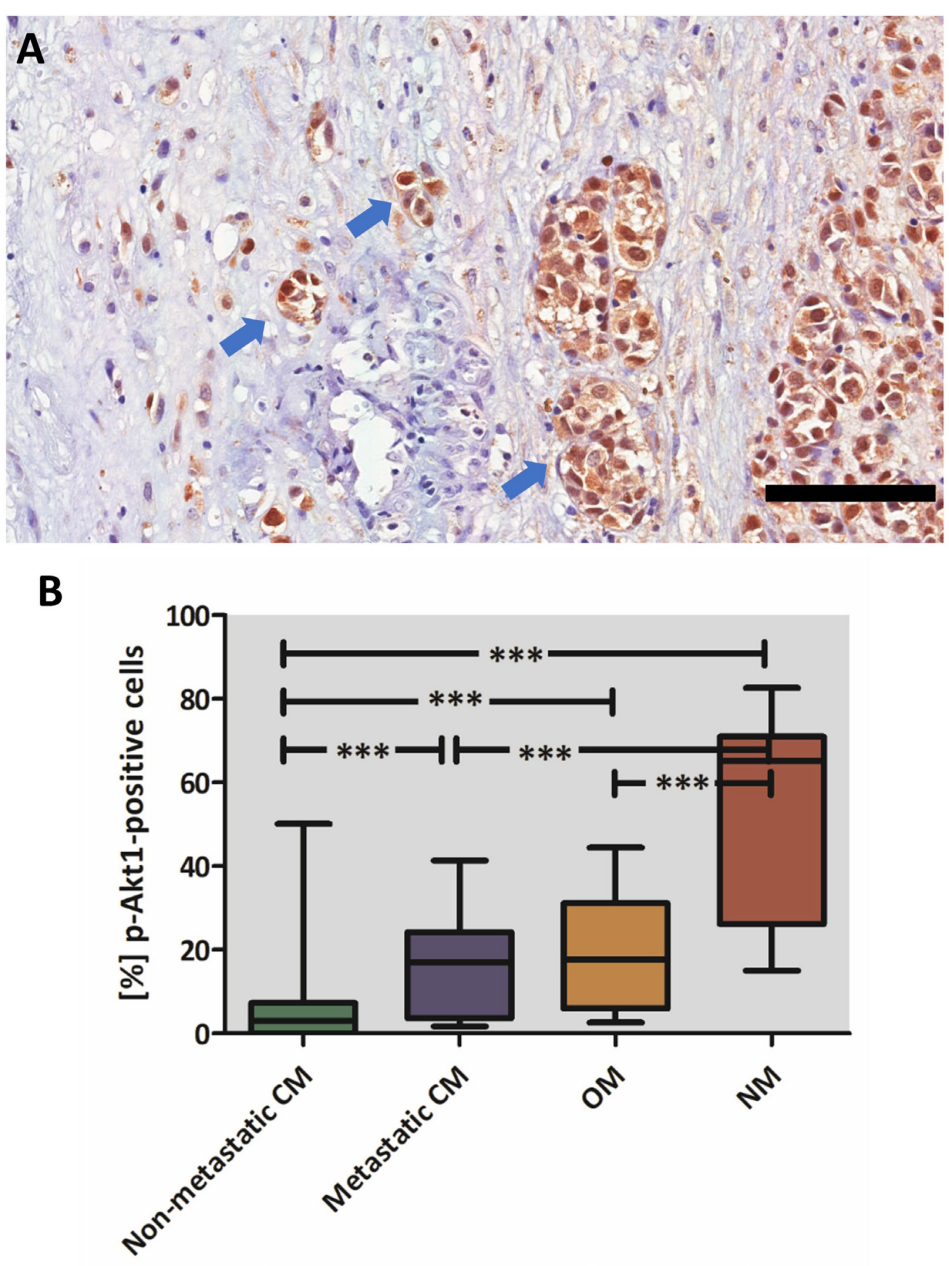
p-Akt1 expression in invasive cells and in different subgroups of melanomas (**A**) Invasive cells strongly express p-Akt1 in the nucleus of a case of sinonasal melanoma. (**B**) Graphical representation of the median, minimum, and maximum scores of p-Akt1-nuclear positivity in the different subgroups of melanomas (^***^indicates statistically significant difference between the groups, ANOVA test, *P* value < .00001). The scale bar represents 100 μm.

The p-Akt1 expression was predominantly detected in the nucleus, and non-metastatic cutaneous melanomas have a mean of 5.6% of the nuclei positive (ranging from 0% to 50.15%). Primary metastatic cutaneous melanomas have a mean of 15.7% (ranging from 1.7% to 41.3%). Cutaneous melanomas with low and high p-Akt1-nuclear expression are illustrated in Figure 1. The representative photomicrographs for p-Akt1 expression in oral and sinonasal melanomas are illustrated in Figures 2 and 3, respectively. Oral melanomas have scores of p-Akt1-positivity (mean = 19.45%, ranging from 2.6% to 44.4%) similar to the cutaneous melanomas with metastatic history. The sinonasal melanomas demonstrated a higher number of nuclei positive for p-Akt1 (mean = 56.15%, ranging from 15.1% to 82.6%), statistically different from the other subtypes of melanomas analysed in this cohort (Figure 4). The nuclear p-Akt1-expression was closely related with invasive cells in all tumours, as illustrated in Figure 4.

## DISCUSSION

The poor prognosis of patients with mucosal melanomas and their ineffective treatment options evidence that these tumours have a distinct biological signature, which is different from the other subtypes of melanomas [5, 10, 19]. Although some studies have extensively addressed the clinicopathological profile of the mucosal melanomas and their immunoprofile, these tumours have lacked predictive markers for tailored treatment [20–24]. Currently, the insufficient knowledge regarding molecular pathways that are selectively activated in different melanomas subtypes ensures comparative studies in mucosal and cutaneous melanomas. Herein, the authors proposed this comparative study to assess the p-Akt1 prognostic value in a series of cutaneous, oral, and sinonasal melanomas.

We used the AJCC system [25] for cutaneous melanomas staging. The AJCC-stage of cutaneous melanoma’s patients significantly affects the outcome, indicating that it is a reproducible method for suggesting the prognosis and therapeutic options. In this study, we also used the AJCC 7th edition staging system for the mucosal melanomas. This system is more useful in prognosis prediction than the Ballantyne’s staging system, which is mostly applied [19]. However, we used a dichotomized model (III/IV AJCC-stages), and the AJCC-staging had no impact on survival rates for oral melanoma patients, in the multivariate model. The relatively small number of cases, one limitation of our study, may explicate this finding. However, as mucosal melanomas of the head and neck are very rare tumours, the authors have provided novel information regarding sixty-five patients with oral and sinonasal melanomas.

Melanomas with predominant epithelioid morphology have been considered tumours of poor prognosis, as these neoplasms have greater DNA ploidy abnormalities when compared with other cellular morphologies in cutaneous melanomas [26]. Interestingly, in the current study, the epithelioid cellular morphology was found to be associated with a poor prognosis in oral melanomas, and it is in accordance with the findings from previous studies [27]. We also demonstrated a statistically significant association of the epithelioid cellular morphology with p-Akt1 overexpression, indicating a probable correlation between these two features. On the other hand, in sinonasal melanomas, only the undifferentiated cell morphology have been reported to confer poorer prognosis [28]. However, in this study, the spindle cell morphology was associated with worse prognosis for sinonasal melanoma patients.

The protein kinase B is serine/threonine-specific protein kinase involved in several biological activities in a wide range of cells, such as glucose metabolism and apoptosis [29]. To date, three isoforms of this protein have been well-described, AKT1, AKT2, and AKT3 [30]. AKT1 is widely documented for its activity in cellular metabolism of several human cancers [29] and when phosphorylated, p-Akt1 is described as playing an important role in the redox modulation of cell cycle progression [31]. On the other hand, Akt2 seems to play a critical role in cell proliferation through increased glycogen synthesis [32] and Akt3 expression is reported in normal tissues, including the brain, heart, kidney, and fat [33].

Previous studies have demonstrated that p-Akt1-nuclear expression is closely associated with worse prognosis in breast [12], gastric [13], and oesophageal squamous carcinoma [14]. In opposition, it is associated with a favourable outcome in patients with pancreatic cancer [34]. The tumorigenic activity of p-Akt1 has also been investigated in several tumours [35–37] and in the context of tumour cells, Akt1 upregulates cell proliferation, invasion, and migration [38, 39]. Contrastively, its deletion prevents lung tumorigenesis in mice models [40]. One previous study shows the putative role of p-Akt1 in metastatic cutaneous melanomas [41]. In this study, the nuclear p-Akt1 expression was associated with the presence of distant metastasis in patients with cutaneous melanomas. In fact, several studies have addressed the role of p-Akt1 in cancer progression and metastasis [42], including its important part in the development of melanoma metastases in a mice model [18]. In addition, many studies have demonstrated the role of Akt1 in metastases development in several malignancies, such as colorectal [43] and lung [44] cancers. Although a correlation between p-Akt1 expression and the metastatic status in cutaneous melanomas have been found for these tumours, the p-Akt1 showed prognostic values only in the univariate model, but it was lost in the multivariate model. In addition, p-Akt1 expression was correlated with important clinicopathologic features in the cutaneous melanomas, such as Breslow’s thickness, Clark’s level, mitotic rate, and AJCC-stage, indicating that p-Akt1 is correlated with melanoma tumorigenesis and affects the clinical outcome of these patients, emerging as a possible target for personalized therapies.

For oral and sinonasal melanomas, univariate and multivariate analysis identified p-Akt1 as an independent prognostic factor. Our results also showed that p-Akt1 expression was significantly higher in sinonasal melanomas than in oral and cutaneous melanomas. Taken together, these data support the hypothesis that sinonasal melanomas have distinct molecular signature and biological behaviour from its cutaneous counterpart. Importantly, we have demonstrated by immunohistochemistry that p-Akt1 is overexpressed in the nuclei of tumour cells and is closely correlated with poor outcome in a subset of mucosal melanomas, whereas the cutaneous melanomas demonstrated lower p-Akt1 expression compared with the oral and sinonasal melanomas. In fact, several studies have addressed the differences concerning etiologic factors and molecular pathways involved in the pathogenesis of cutaneous *vs* mucosal melanomas [3–5, 45]. For example, c-KIT aberrations were reported in tumours that have no correlation with chronic sun exposure (mainly mucosal melanomas). In opposition, the cutaneous counterpart frequently harbours mutations in the BRAF gene, given the fact that chronic sun exposure is important for its pathogenesis [9, 46]. It has been previously demonstrated that c-KIT is a key molecule for p-Akt1 activation through PI3K phosphorylation [46]. Therefore, particularly in mucosal melanomas, a correlation between p-Akt1 overexpression and c-KIT mutation may help to understand how these pathways are important for melanoma pathogenesis and patient outcomes.

In the cohort of sinonasal melanomas analysed in this study, p-Akt1 overexpression showed a correlation with higher number of mitosis, presence of vascular invasion, and with spindle and undifferentiated cellular morphology. These findings provide new insights about the probable role of p-Akt1 in the aggressive genotype and phenotype of the sinonasal melanomas. In these particular tumours, necrosis was associated with overall survival in one previous report [47]. However, in this study’s cohort, necrosis has not demonstrated prognostic predicting value, corroborating with other previous studies [48, 49]. No correlation between p-Akt1 and necrosis was observed in this study.

Overall, the treatment of choice for both mucosal and cutaneous melanoma is the wide surgical resection [50–52]. Although it has been demonstrated that melanomas are not radiosensitive, some tumours have been treated with adjuvant radiotherapy, and the use of these combined therapies are not standardized [10]. Regarding the mucosal melanomas, approximately 30% of the patients from the sample with oral melanomas and 50% of the patients with sinonasal melanomas received chemotherapy or radiotherapy as adjuvant treatment. Nevertheless, no differences in the cancer-specific survival were observed in both groups. The use of adjuvant therapies with surgical resection still needs to be investigated as a option to treat patients with mucosal melanomas [53].

In conclusion, the results of the current study demonstrated that p-Akt1 overexpression is an independent prognostic marker in mucosal melanomas and is significantly up-regulated in sinonasal melanomas. As both mucosal and metastatic cutaneous melanomas showed high frequency of p-Akt1 expression, our findings suggest that mucosal melanomas have a biological behaviour that is similar to the one identified in aggressive cutaneous melanomas.

## MATERIALS AND METHODS

### Ethical issues

The study protocol was approved by the National Commission for Ethics in Research (CONEP-Brazil, CAAE: 72077517.1.0000.5418) and all procedures were in accordance with the Declaration of Helsinki.

### Patient samples and data collection

Cutaneous, sinonasal, and oral samples from the surgical samples of patients with melanoma who were referred to four Brazilian and Guatemalan cancer centers since 1997 were collected and further analysed. Melanoma diagnoses were confirmed by three experienced pathologists based on the microscopic examination of haematoxylin–eosin–stained slides, and on the S-100 protein, MART-1/Melan A and gp-100 (HMB-45) expressions.

In total, 209 samples, including cutaneous (*n* = 144), oral (*n* = 34) and sinonasal (*n* = 31) melanomas. The first patient was included in May 1997 and the last patient in February 2016. Surviving patient follow-up was censored on September 10, 2017. Clinical data were collected from the patient’s medical records which included age, sex, tumour location, metastases (presence/absence and site), clinical-stage, treatment options, and follow-up data. Tumour stage was determined according to the seventh edition of the staging manual of the American Joint Committee on Cancer [25].

### Tissue microarray construction

For construction of the 7 tissue microarray blocks, a previously reported method was followed [41, 54]. Duplicate cores of 2 mm^2^ were collected from the original blocks of cutaneous, oral, and sinonasal melanoma patients.

### Immunohistochemistry (IHC) and digital scoring

IHC manual technique was performed using 3-mm thick formalin-fixed paraffin-embedded melanomas sections mounted on silane-coated glass slides. An anti-Phospho-Akt1 (clone D7F10, 1:100 dilution; Cell Signalling, Danvers, Massachusetts, USA) was used. The antigen detection was achieved using the ADVANCE^™^/HRP (code K406889-2; Dako, Carpinteria, CA, USA), revealed with the 3,3′-diaminobenzidine-tetrahydrochloride chromogen and counterstained with Carazzi’s haematoxylin.

All slides were scanned into high-resolution images with Aperio Scanscope CS Slide Scanner (Aperio Technologies Inc., Vista, California, USA). In order to calculate the scores of positivity expression of p-Akt1, digital analyses were performed, as previously described [41, 55]. Nuclear p-Akt1 expression was scored based on the percentage [%] of positive nuclei, assessed digitally with the Nuclear Algorithm (Aperio Technologies Inc.).

### Statistical analyses

For the statistical analyses, all collected data were recorded in a password-protected computer database. All statistical analyses were carried out using the SPSS software, version 18.0 (SPSS Inc., Chicago, IL, USA). In the univariate model, the cancer-specific survival (CSS) (defined as the time between the start date of the treatment and the date of death due the disease) and disease-free survival (DFS) (defined as the time between treatment and recurrence) were estimated comparing the Kaplan–Meier survival curves using the log-rank test. The multivariate Cox regression was performed for prognostic significance determination of the p-Akt1 expression Index. For both univariate and multivariate models, the association between the variables age, sex, primary tumour site and p-Akt1 expression and CSS and DFS were evaluated. *p*-value ≤ .05 was considered statistically significant.

## SUPPLEMENTARY MATERIALS TABLES



## Abbreviations

CM: cutaneous melanomas
OM: oral melanomas
SNM: sinonasal melanomas
HR: Hazard Ratio
AJCC: American Joint Committee on Cancer
p-Akt1: Phosphorylated Akt1, or protein B kinase 1
IHC: immunohistochemistry
CSS: Cancer-specific survival
DFS: disease-free survival
