# Supplementary material for: Phosphorylated Akt1 expression is associated with poor prognosis in cutaneous, oral and sinonasal melanomas
Source: Oncotarget. 2018 Dec 18;9(99):37291–304. doi: 10.18632/oncotarget.26458 (PMC6324666; doi:10.18632/oncotarget.26458)
Supplement: Supplementary file 1 [file oncotarget-09-37291-s001.pdf]

Phosphorylated Akt1 expression is associated with poor prognosis in cutaneous, oral and sinonasal melanomas

SUPPLEMENTARY MATERIALS

Supplementary Table 1: Clinicopathologic features of 144 patients with cutaneous melanomas

Factors	Category	Frequency *n* (%)
Gender	Female	71 (49.3)
	Male	73 (50.7)
Age (years)	Range; median	20–88; 56
	Mean (SD)	56 (15.7)
Site	Head and neck	22 (15.3)
	Trunk	64 (44.4)
	Upper limbs	18 (12.5)
	Lower limbs	40 (27.8)
T-stage	*In situ*	15 (10.4)
	I	51 (35.4)
	II	42 (29.2)
	III	23 (16.0)
	IV	13 (9.0)
Clark's level	I and III	47 (32.6)
	III, IV and V	97 (67.4)
Ulceration	Present	61 (42.4)
	Absent	83 (57.6)
Mitotic rate (number/mm²)	<3	60 (41.7)
	≥3	84 (58.3)
Breslow's thickness (mm)	<1.55	72 (50)
	≥1.55	72 (50)
Distant metastasis	Present	34 (23.6)
	Absent	110 (76.4)

Supplementary Table 2: Clinicopathologic features of 34 patients with oral melanomas

Factors	Category	Frequency *n* (%)
Gender	Female	16 (47.1)
	Male	18 (52.9)
Age (years)	Range; median	19–89; 47
	Mean (SD)	52 (19.5)
Site	Palate	15 (44.1)
	Alveolus	5 (14.7)
	Other	14 (41.2)
Cell morphology	Epithelioid	22 (64.7)
	Non-Epithelioid	12 (35.3)
Vascular invasion	Present	13 (38.2)
	Absent	21 (61.8)
Neural invasion	Present	8 (23.5)
	Absent	26 (76.5)
Necrosis	Present	10 (29.4)
	Absent	24 (70.6)
Mitotic rate (number/mm^2)	<1	14 (41.2)
	≥ 1	20 (58.8)
Treatment	Only surgery	22 (64.7)
	Surgery plus chemotherapy or radiotherapy	11 (32.4)
	Other/missing	1 (2.9)
Clinical Stage	III	9 (26.5)
	IVa	16 (47.1)
	IVb	4 (11.8)
	IVc	5 (14.7)

Supplementary Table 3: Clinicopathologic features of 31 patients with sinonasal melanomas

Factors	Category	Frequency *n* (%)
Gender	Female	15 (48.4)
	Male	16 (51.6)
Age (years)	Range; median	24–82;58
	Mean (SD)	55 (17)
Site	Nasal cavity	13 (41.9)
	Maxillary sinus	12 (38.7)
	Rhinopharynx	6 (19.4)
Cell morphology	Epithelioid	9 (29.0)
	Fusiform	13 (41.9)
	Plasmacytoid	4 (12.9)
	Undifferentiated	5 (16.1)
Vascular invasion	Present	9 (28.1)
	Absent	22 (71.9)
Neural invasion	Present	4 (12.9)
	Absent	27 (87.1)
Necrosis	Present	13 (41.9)
	Absent	18 (58.1)
Mitotic rate (number/mm^2)	<1	20 (64.5)
	≥1	11 (35.5)
Treatment	Only surgery	13 (41.9)
	Surgery plus chemotherapy or radiotherapy	17 (54.8)
	Other/missing	1 (3.3)
Clinical stage	III	9 (29.0)
	IVa	11 (35.5)
	IVb	4 (12.9)
	IVc	7 (22.6)
